# Complete Genome Sequences of Fish Pathogenic *Weissella ceti* Strains WS74 and WS105

**DOI:** 10.1128/genomeA.01014-14

**Published:** 2014-10-16

**Authors:** H. C. P. Figueiredo, C. A. G. Leal, F. A. Dorella, A. F. Carvalho, S. C. Soares, F. L. Pereira, V. A. C. Azevedo

**Affiliations:** a AQUACEN, National Reference Laboratory for Aquatic Animal Diseases, Ministry of Fisheries and Aquaculture, Federal University of Minas Gerais, Belo Horizonte, Brazil; b Laboratory of Cellular and Molecular Genetics, Institute for Biological Science, Federal University of Minas Gerais, Belo Horizonte, Brazil

## Abstract

We describe here the genome sequencing and annotation of *Weissella ceti* strains WS74 and WS105, isolated from diseased rainbow trout in Brazil. The two genomes were sequenced with an Ion Torrent personal genome machine (PGM) using a fragment library. The genomes of strains WS74 and WS105 consist of circular chromosomes 1,389,513 bp and 1,390,396 bp long, respectively, both presenting a G+C content of 40.75%.

## GENOME ANNOUNCEMENT

The *Weissella* species belong to the group of lactic acid bacteria (LAB) and have been isolated from a broad variety of nutrient-rich environments (1) and animals (2–6). Recently, the first species associated with diseased fish and beaked whales was reported, named *Weissella ceti* (7, 8). *W. ceti* has been isolated from outbreaks of hemorrhagic disease in rainbow trout in Brazil (9, 10). Although genome data for *W. ceti* strains WS08 and NC36 exist, comparative genome analyses for discovering new virulence factors are partially impaired by the draft status of *W. ceti* NC36 (11). We sequenced *W. ceti* strains WS74 and WS105 isolated from rainbow trout in Brazil during different outbreaks and years and presenting distinct pulsed-field gel electrophoresis (PFGE) types (9).

*W. ceti* strains WS74 and WS105 were thawed, streaked onto Man Rogosa and Sharpe (MRS) agar (Sigma-Aldrich, USA), and incubated at 25°C for 48 h. The DNA was extracted with a Maxwell 16 DNA purification kit using a Maxwell 16 instrument (Promega, USA). Extracted DNA molecules were quantified with a Qubit 2.0 fluorometer (Life Technologies, USA), and the DNA integrity was assessed using TapeStation (GE, USA). The DNA was sequenced in a personal genome machine (PGM) Ion Torrent sequencing system (Life Technologies, Carlsbad, CA) using a fragment library with an Ion PGM 200 sequencing kit.

The genome sequencing of *W. ceti* WS74 yielded genome coverage of ~210×. An initial assembly of WS74 with Mira 4.0 (12) and Newbler 2.9 resulted in 120 and 19 contigs with *N*_50_s of ~68 kb and ~131 kb, respectively. For the genome of *W. ceti* WS105, genome coverage of ~296× was obtained, and the initial assembly with Mira and Newbler resulted in 473 and 20 contigs with *N*_50_s of ~24 kb and ~162 kb, respectively. For both strains, the software programs CONTIguator 2.0 (13) and FGAP 1.7 (14) were used for recursive and reference-based (WS08 strain) overlapping of contigs. The remaining gaps were solved using recursive and reference-based read mapping with CLC Genomics Workbench version 7.0 (CLC bio, Germantown, MD).

Automatic gene and pseudogene prediction and annotation were performed using the software PROKKA version 1.7 (15) and RAST server version 4.0 (16). All putative frameshifts were manually curated based on the coverage and quality of the given base using genome mapping. The genome of *W. ceti* WS74 comprises 1,389,513 bp with a G+C content of 40.75% and contains 1,438 genes, which represent 1,338 coding sequences (CDSs), 3 pseudogenes, 1 tmRNA, 77 tRNAs, and 19 rRNAs. The genome of *W. ceti* WS105 has 1,390,396 bp with a G+C content of 40.75% and harbors 1,433 genes, which are composed of 1,336 CDSs, 1 tmRNA, 71 tRNAs, and 18 rRNAs. The genome sequences of the 2 strains of *W. ceti* described here open new doors for genome comparison of this bacterium, which will help in elucidating the mechanisms that drove the emergence of an LAB as a new fish pathogen.

### Nucleotide sequence accession numbers.

The whole-genome projects of *W. ceti* strains WS74 and WS105 have been deposited at Genbank under the accession numbers CP009223 and CP009224, respectively.
